# Spatio-Temporal Patterns of Barmah Forest Virus Disease in Queensland, Australia

**DOI:** 10.1371/journal.pone.0025688

**Published:** 2011-10-13

**Authors:** Suchithra Naish, Wenbiao Hu, Kerrie Mengersen, Shilu Tong

**Affiliations:** 1 School of Public Health, Queensland University of Technology, Brisbane, Queensland, Australia; 2 School of Population Health, The University of Queensland, Brisbane, Queensland, Australia; 3 Mathematical Sciences, Queensland University of Technology, Brisbane, Queensland, Australia; Kenya Medical Research Institute - Wellcome Trust Research Programme, Kenya

## Abstract

**Background:**

Barmah Forest virus (BFV) disease is a common and wide-spread mosquito-borne disease in Australia. This study investigated the spatio-temporal patterns of BFV disease in Queensland, Australia using geographical information system (GIS) tools and geostatistical analysis.

**Methods/Principal Findings:**

We calculated the incidence rates and standardised incidence rates of BFV disease. Moran's *I* statistic was used to assess the spatial autocorrelation of BFV incidences. Spatial dynamics of BFV disease was examined using semi-variogram analysis. Interpolation techniques were applied to visualise and display the spatial distribution of BFV disease in statistical local areas (SLAs) throughout Queensland. Mapping of BFV disease by SLAs reveals the presence of substantial spatio-temporal variation over time. Statistically significant differences in BFV incidence rates were identified among age groups (χ^2^ = 7587, df = 7327,p<0.01). There was a significant positive spatial autocorrelation of BFV incidence for all four periods, with the Moran's *I* statistic ranging from 0.1506 to 0.2901 (p<0.01). Semi-variogram analysis and smoothed maps created from interpolation techniques indicate that the pattern of spatial autocorrelation was not homogeneous across the state.

**Conclusions/Significance:**

This is the first study to examine spatial and temporal variation in the incidence rates of BFV disease across Queensland using GIS and geostatistics. The BFV transmission varied with age and gender, which may be due to exposure rates or behavioural risk factors. There are differences in the spatio-temporal patterns of BFV disease which may be related to local socio-ecological and environmental factors. These research findings may have implications in the BFV disease control and prevention programs in Queensland.

## Introduction

Mosquito-borne arboviral diseases are a common but frequently neglected public health problem in many parts of the world [1]. Recently, arboviral activity has increased due to the changes in wetlands, land practices, and irrigation practices, which often result in massive mosquito breeding and disease outbreak [2], [3], [4]. Of the arboviruses important in human infection, Barmah Forest virus (BFV) is the second most common (after Ross River virus) disease in Australia [5], [6]. BFV belongs to the *Alphavirus* genus and *Togaviridae* family [7], [8]. Barmah Forest virus was first isolated in 1974 from *Culex annulirostris* mosquitoes collected in the Barmah Forest near the Murray River in northern Victoria [9], and simultaneously from mosquitoes collected in southwest Queensland [10]. The genera *Culex* and *Aedes* are primarily involved in the transmission along the inland and coastal areas in Queensland, respectively [11], [12], [13].

BFV disease can be detected by serological tests either by a rise in antibody titre to the BFV or detection of BFV-specific IgM or isolation of BFV from clinical material [14]. The symptoms of the disease include fever, skin rash and body and muscle pains [15], [16], [17]. Some or all of these symptoms may be present. It has no specific treatment. No known cases of death have occurred from this disease. It affects people of all ages irrespective of gender. BFV disease is caused by pathogens transmitted by animals [18] and some studies [19] reveal that kangaroos and wallabies are reservoir hosts , while other studies [20] indicate that birds are involved in the transmission of the disease. However, possums, cats and dogs are unlikely to be important hosts [21], [22].

BFV is a notifiable disease and the cases have been documented in every state and territory across Australia [23]. For example, during the period 1995–2008, a total of 15,592 BFV cases were recorded in Australia. Of these, the largest number of cases was from Queensland (N = 8,050) [24]. There is a trend of increasing BFV cases in Australia over recent years [27]. Possible reasons for this increase may include urban developments and socio-ecologic changes such as human movement and changes in life-style activities [25], [26].

The transmission dynamics of BFV disease are affected by various biotic (e.g. abundance and distribution of mosquitoes and susceptible vertebrate hosts) and abiotic factors (e.g. temperature) [4], [8], [12], [13], [27]. Other human related factors such as behaviour and immunity are also involved in the transmission [27]. A few studies indicate that climate variables such as temperature, rainfall and relative humidity are potentially involved in the transmission of BFV disease [28], [29]. However, the exact roles of climatic, ecological and socio-environmental variables in BFV disease transmission are not yet thoroughly explored.

For a mosquito-borne disease, it is important to understand the spatial and temporal characteristics of its natural transmission. Geographic information systems (GIS) have been widely adopted in the spatial studies of mosquito-borne diseases [30], [31], [32], [33]. Geo-coding or geo-referencing is the basic GIS procedure, which refers to a spatial location of a data layer which is defined by a known co-ordinate reference system such as latitude and longitude, the reference units (eg, meters) and the coordinating positions of the boundary of the mapped area [34]. GIS has an important role in surveillance and control of the mosquito-borne diseases as it is possible to analyse factors associated with the disease through the geo-coding processes [35]. GIS facilitated maps are useful for the identification of spatially and temporally intensified infection areas and potential high-risk populations [35]. The visualized information presented in different types of maps based on GIS [36], [37], [38] enable simultaneous observation of both the attribute and geographical relationships [39]. Maps also help public health officials to communicate with the public and policy makers about complex information in an easily understood format [39].

GIS can provide not only an avenue to improve our understanding of distribution patterns of BFV disease, but also an environmentally and socially informed platform to develop early warning systems towards control and prevention of BFV disease. Hence, in this study, we aimed to examine the spatio-temporal patterns of BFV disease in Queensland, Australia using GIS tools and geostatistical analysis.

## Methods

### Study area

Queensland is the third high-density populated state in Australia (after New South Wales and Victoria), occupying a total area of 1,723,936 km^2^ with a total population of 4.29 million people (20% of Australia's total) and is the fastest growing state with 1,500 people moving in per week in 2009 [40]. There are 11 statistical divisions (SDs) and 74 Local Government Areas (LGAs), consisting of 478 Statistical Local Areas (SLAs) [41]. The smallest geographical unit is SLA in Australian census data. Queensland is selected as the study area as the average annual incidence of Queensland is three times higher (incidence rate = 29.0/100,000 population) than the national average annual incidence (incidence rate = 9.8/100,000 population) in 2008 [42]. Climate generally ranges from the temperate and densely populated southeast to the tropical, sparsely populated Cape York Peninsula in the north.

### Data collection

#### Ethics statement

The study was approved by the Data custodians, Human Research Ethics Committee under Chapter 6, Part 4, Section 280 of the Public Health Act 2005, Communicable Diseases Branch (CDB) in the Queensland Health and following the ethical considerations of the Research Ethics Unit, Queensland University of Technology.

#### BFV disease data

Since BFV is a notifiable disease, all positive test results are required to be reported by laboratories to the Queensland Health, by the Public Health Act 2005 legislation [42]. These records are archived by the Data custodians, CDB in the Queensland Health under the National Notifiable Disease Surveillance System (NNDSS) Scheme. The NNDSS was established in 1990 under the auspices of the Communicable Diseases Network Australia. Vector-borne diseases notified to the NNDSS include mosquito-borne diseases caused by alphaviruses, such as BFV and RRV. Under NNDSS scheme, BFV cases were made to Queensland Health under the provisions of the Public Health Act 2005 [42].

We obtained computerised and non-identifiable BFV disease notification data (data that do not contain any identifiers such as name, street number or Medicare number) from January 1993 to December 2008 (16 years) for the study area in Microsoft Excel format from the Data custodians, CDB, Queensland Health [42]. No individual will be personally identifiable in any results arising from this study. BFV disease data included date of notification, age, gender and residential address (street/road name and suburb name, i.e., location, post code of residence).

#### Population data

Population data for the SLAs for the national census years 2001 and 2006 were obtained from the Australian Bureau of Statistics [41]. The population data (as those from basic community profiles) from SLA level were used for epidemiological surveillance. For the other years during 1993 to 2008, the annual population data were estimated based on the average annual changes for the years considering the population growth between 2001 and 2006 [40].

#### Geocoding

We aggregated BFV disease notification data from postcode to SLA level using MapInfo Professional [43]. A total of 9,267 cases were supplied by Queensland Health. Of these, 6,788 (73.3%) were geocoded after three attempts. In the first attempt, a total of 4,424 (47.7%) cases were geocoded using street/ suburb name. In the second attempt, a total of 1,567 (17%) cases were geocoded using street name and post code of residence. In the first attempt, 797 (8.6%) cases were geocoded by manually correcting the spelling errors of street/suburb name. A total of 2,479 (26.7%) cases were not geocoded mainly due to incorrect spelling or entering the street/suburb name wrongly and those were unable to correct manually.

### Data analysis

The study period was divided into four time periods, with four years in each time period for the ease of the analysis: Period 1: 1993-1996, Period 2: 1997–2000, Period 3: 2001–2004 and Period 4: 2005–2008. Population data for each period were attached to the maps and these were used as the denominator in the computation of incidence rates. Period-wise distribution maps were produced on BFV cases and incidence rates by SLA. MapInfo Professional was used to produce the final outputs as tabular forms and maps.

#### Spatial and temporal analyses

To investigate the spatial and temporal patterns of BFV disease and to determine the risk of BFV disease, monthly incidence rates were calculated at both SLA and state level. Age-wise incidence rates were also calculated from the total number of BFV cases notified in each age group for each SLA in different time periods, divided by the respective total person-years and then multiplied by 100,000.

The incidence rate can be expressed as: 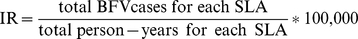



Differences between estimates of incidence rates for age and gender were tested using chi-square analyses.

Age and gender standardised incidence rates (SIRs) were calculated for each SLA, using the direct method (based on Queensland population as a “reference”), adjusted for differences in the age and gender distribution [44]. For example, BFV disease was high among 40–49 year old age group so a SLA with a higher proportion of this age group would have a higher overall incidence rate of BFV disease. In order to determine which SLA had a higher incidence rate regardless of age and gender distribution, the rates were age and gender standardised. The standardisation of rates and corresponding confidence intervals (CI) took population size into account, thus avoiding possible bias associated with small counts and small sub-populations. Moreover, the reported results included the uncertainties of SIRs (ie, 95% CI). *A*n example using actual data from the study sample only including six SLAs in Queensland is shown in Appendix S1.

The equation for calculating SIR is:
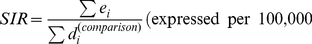



Where 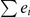 is the total number of expected cases generated using the reference population rates for each SLA; 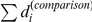 is the total population in the comparison group.

An SIR of 100,000 indicates that the number of BFV cases observed in the population evaluated is equal to the number of BFV cases expected in the comparison with “reference” population. In this study, we calculated SIR for the entire Queensland state including 478 SLAs using the same method.

To further examine spatial differences among SLAs, the SIR estimates were mapped and 95% confidence intervals (CIs) [45] were calculated in each SLA. A significant difference between the observed and expected number of cases is asserted if the CI does not contain zero. Differences between observed and expected number of cases estimates for age and gender were tested using chi-square analyses.

#### Spatial analysis

Spatial analysis comprised of four procedures: 1) evaluation of spatial autocorrelations, 2) semi-variogram modeling, 3) interpolation of SIR values based on kriging and 4) interpolation of incidence rates based on inverse distance weighting (IDW).

#### Spatial autocorrelations

The global Moran's *I* test statistic was used to assess the presence of significant spatial autocorrelation of BFV disease incidence rates in four different periods of 1993–1996, 1997–2000, 2001–2004 and 2005–2008 using GeoDa software [46]. Moran's *I* ranges from −1 to 1: a value close to 0 indicates spatial randomness while a positive value indicates positive spatial autocorrelation, vice versa. Statistical significance was tested using randomisation based on 999 permutations. The weight distance matrix, essential for the computation of spatial autocorrelation statistics, was based on Queen contiguity and Euclidean distance [46].

#### Semi-variogram analysis

We used semi-variogram modeling analysis [47], [48] to explore the spatial structure and spatial autocorrelation of SIRs of BFV disease and age. The underlying assumption of the variogram is that two observations close together are more similar than those further apart.

The semi-variogram is a plot of the semi-variance against lag distance. If the semi-variance is markedly small for low values of lag distance, it is considered as an indication of spatial autocorrelation, i.e., values at short distance from each other are more alike than those at larger distances [49], [50]. The best-fit semi-variogram model was identified by using Vertical Mapper within MapInfo Professional [51].

#### Kriging interpolation

A map of kriged SIR values was created based on the best-fit semi-variogram model to better visualise the distribution of spatially related patches of BFV disease. The kriged SIR values were obtained using the interpolation method in Vertical Mapper within MapInfo Professional [51].

Geostatistical approaches, such as kriging methods, are designed to model the spatially dependent local component [47], [48]. This approach describes the spatial dependence through a semi-variogram model. This model is then applied locally to account for the spatially dependent local variation. When estimating the value of a variable at a location, this approach does not look into explanatory factors at the location. Rather, it uses values of the variable at adjacent locations for the estimation.

Since the semi-variogram describes the spatial dependency between the observed measurements as a function of the distance between them, it allows us to estimate the SIR value of BFV disease at any point from the observed data.

#### Inverse distance weighing (IDW) interpolation

We used the IDW interpolation method to map the interpolated incidence rates of BFV disease across the state. This is because mapping the spatial distribution of BFV disease and potential risk areas requires converting points into surfaces. The IDW interpolation [52] technique is commonly used in GIS programs for producing surfaces using interpolation of scatter points and has been employed in other analyses of mosquito-borne diseases [53], [54], [55].

IDW weighs the contribution of each input (control) point by a normalized inverse of the distance from the control point to the interpolated point. The IDW interpolation method assumes that each input point has a local influence that decreases with distance. It weighs the points closer to the processing points more than those far away [56].

We grouped the dataset into four categories, each consisting of four-year period between 1993–2008. For each period, we spatially interpolated the incidence rates of BFV disease by applying the IDW procedure using Vertical Mapper within MapInfo Professional.

#### Temporal analysis

To examine temporal patterns, epidemic curves were produced by calculating the annual incidence rate of BFV disease (annual BFV disease cases for each year divided by total population for each year * 100,000 people) and monthly cases of BFV disease during the period 1993–2008. Monthly differences between IR estimates for the overall time period were tested using chi-square analyses.

## Results

### Descriptive analysis

**Figure 1:**
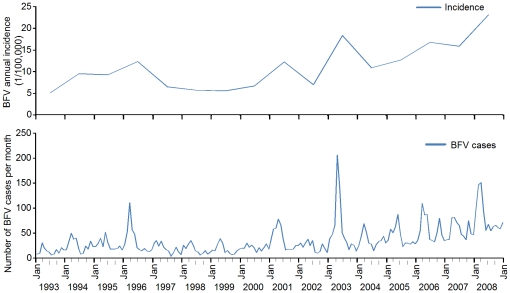
Temporal distribution of BFV disease in Queensland, 1993 to 2008.

Overall, the average number of BFV cases was 417.6 cases per year in Queensland. Figure 1 shows the epidemic patterns of the temporal distribution of incidence rates of BFV disease during 1993–2008 in Queensland. The annual incidence had fluctuated around 5.26 (in 1993) to 22.86/ per 100,000 people (in 2008) (Fig 1). Table 1 shows summary statistics for the incidence rates of BFV disease for the four time periods across Queensland.

**Table 1:** Descriptive statistics of incidence rates of BFV disease in Queensland, Australia, (n = 6,683).

Period	Mean	S.D.	Minimum	Q1*	Median	Q3†	Maximum
1993–1996	31.64	51.33	0.00	0.00	12.80	40.38	420.17
1997–2000	23.45	41.46	0.00	0.00	10.57	32.21	573.07
2001–2004	44.23	56.42	0.00	0.00	27.72	57.01	404.59
2005–2008	65.50	82.86	0.00	0.00	39.98	89.85	731.73

Q1* = first quartile value; Q3† = third quartile value.

**Figure 2:**
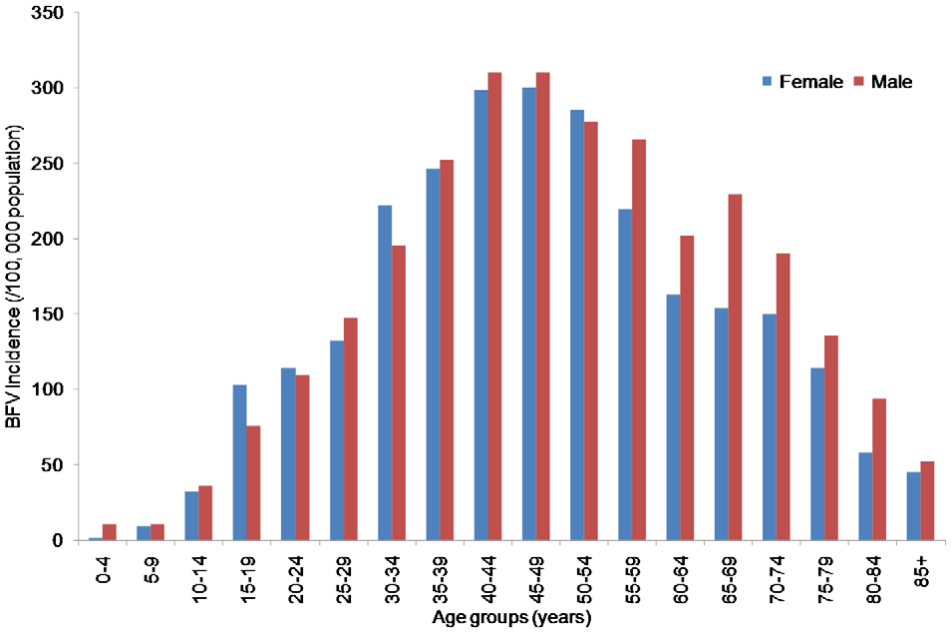
Incidence rates of BFV disease by age and gender in Queensland, 1993–2008.

The median age of the BFV disease patients was 44 years (range<1–98 years). The age and gender distributions are comparable with the last national population census data [45]. Figure 2 shows the annual incidence rate (per 100,000 people) during 1993–2008. The average annual incidence rate increased steadily with increasing age, ranging from 5.96 /100,000 in children aged<10 years to 305.19 among those aged 40–49 years. Slightly more males (52.3%) were affected by BFV disease than females (47.6%). Figure 2 indicates that males of almost all age groups had higher incidence rate compared with females and the difference between age groups and incidence rates was statistically significant (χ^2^ = 7587, df = 7327, p<0.01).

In addition, the incidence rates of BFV disease during 1993–2008 in Queensland indicates a strong seasonal pattern (χ^2^ = 1379, df = 1146 ,p<0.01), peaked in Autumn (i.e., February to May) and diminished in Winter (i.e., July to October).

### Spatial and temporal analyses of BFV disease among SLAs

#### Incidence rates

A total of 435 out of 478 SLAs (91%) in Queensland were notified with BFV cases and their number varied over four periods. Table 2 shows the geographical characteristics, number of SLAs with BFV cases, population sizes for SLAs and population density (Table 2). Period-wise, the SLAs notified with no BFV cases were varied. There were 280 SLAs with BFV cases in 1993–1996 (1164 cases), 265 SLAs in 1997–2000 (859 cases), 347 SLAs in 2001–2004 (1827 cases) and 385 SLAs in 2005–2008 (2833 cases). In all four time periods, there were statistically significant differences between the observed and expected numbers of BFV cases within SLAs (Table 2).

**Table 2:** Characteristics of BFV disease transmission and population growth during 1993 and 2008 (n = 478 SLAs).

Period	No. of SLAs with BFV disease	No. of BFV cases in SLAs	Average QLD Population	Population density (person/km^2^)	p-value (χ^2^)
1993–1996	280	1164	3169386	338313	0.02
1997–2000	265	859	3436366	363398	0.008
2001–2004	347	1827	3763262	396525	0.003
2005–2008	385	2833	4144504	431458	0.002

Overall, the coastal regions had the highest incidence rates: SLAs with highest incidence rates were Miriam Vale (incidence rate  = 1,146/100,000 people) followed by Barcoo, Barcaldine, Noosa (Balance), Weipa, Aramac, Calliope, Redland, Noosa (Tewantin), Douglas, Caloundra, Winton, Railway Estate, Cardwell, Maroochy, Magnetic Island and Carpentaria (incidence rate = 527/100,000 people). Period-wise, Miriam Vale had the highest incidence rate of 731/100,000 people during 2005–2008, followed by 260/100,000 people during 2000–2004. Mostly BFV disease had spread dramatically from north to south and west during 1993–2008. In general, BFV disease recurred for 16 consecutive years along the coastal regions, with the highest number of cases reported in 2008 (n = 982), indicating that BFV disease has become seasonally endemic.

**Figure 3:**
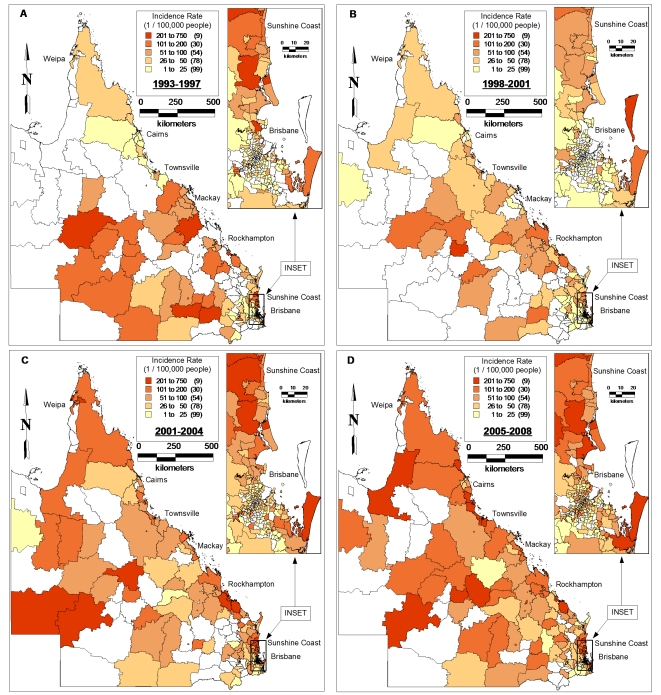
Maps showing the incidence rates of BFV disease by SLA over different periods (A:1993–1996, B:1997–2000, C:2001–2004 and D:2005–2008).

Figure 3 shows substantial variation in the geographic distribution of incidence rates of BFV disease among SLAs in Queensland across four time periods. It indicates that in each of the time periods, most BFV cases occurred in the proximity of coastal areas. However, the incidence rate in each SLA varied during 1993–2008 (Fig 3).

#### Spatial autocorrelation

There was a significant spatial positive autocorrelation of incidence rates of BFV disease for all four periods, with Moran's *I* statistics of 0.2901 (p = 0.001) during 1993–1996, 0.1506 (p = 0.001) during 1997–2000, 0.2685 during 2001–2004 (p = 0.001), and 0.2737 during 2005–2008 (p = 0.001) (Table 3). There was an increase in spatial autocorrelation over the period 1997–2008, reaching the highest value during 2005–2008.

**Table 3:** Spatial autocorrelation analysis for BFV disease in Queensland, 1993–2008.

Period	Moran's *I*	Mean	SD	E[I]	P
1993–1996	0.2901	−0.0043	0.0292	−.0021	0.001
1997–2000	0.1506	−0.0027	0.0259	−.0021	0.001
2001–2004	0.2685	−0.0004	0.0289	−.0021	0.001
2005–2008	0.2737	−0.0017	0.0302	−.0021	0.001

**Figure 4:**
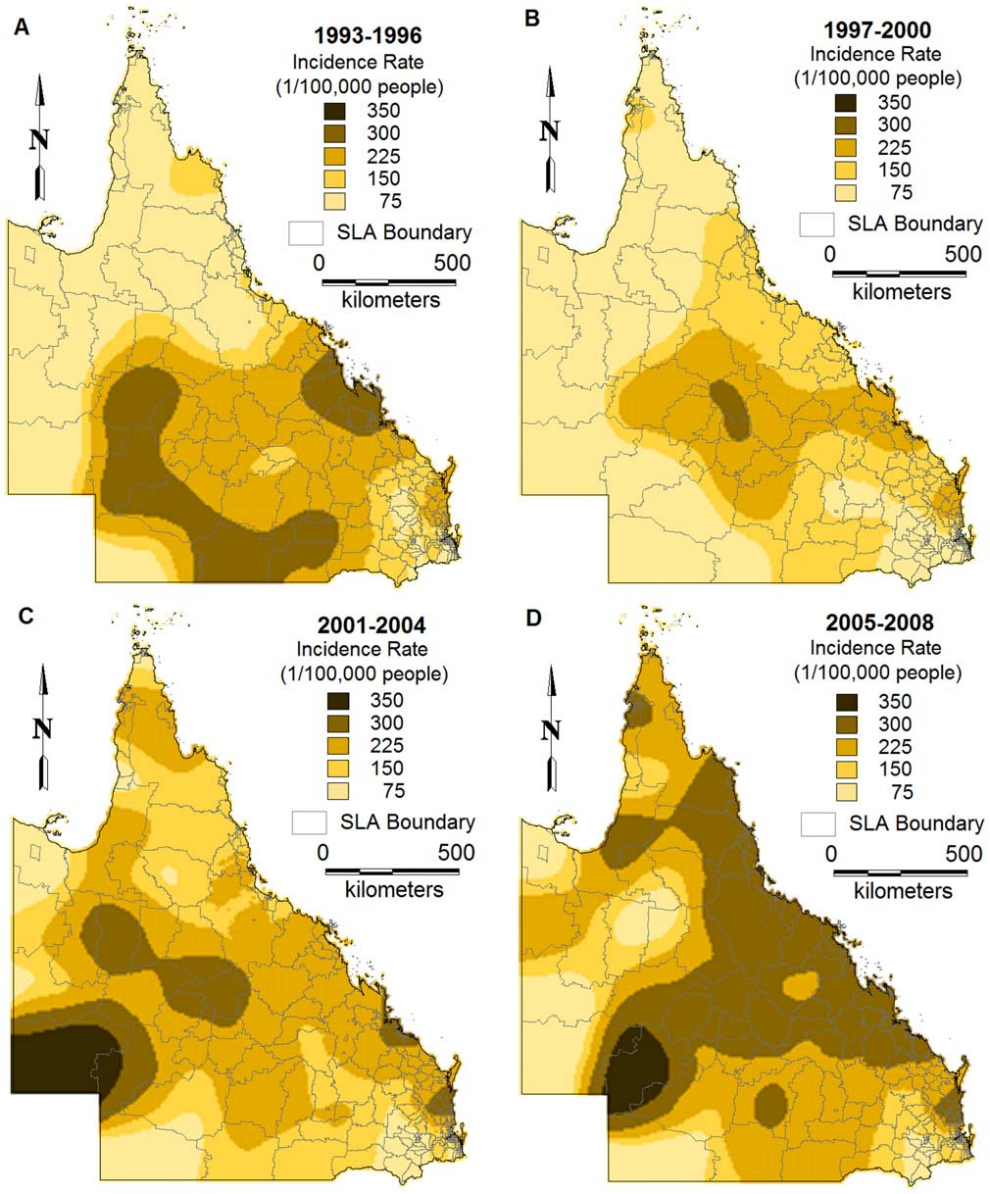
Maps showing the inverse distance weighting interpolated incidence rates of BFV disease by SLA over different periods (A:1993–1996, B:1997–2000, C:2001–2004 and D:2005–2008).

Figure 4 depicts the spatial distribution of interpolated estimates of incidence rates of BFV disease in the four time periods using IDW method. It visually confirms that the incidence rates of BFV disease varied geographically across the state.

#### Standardised incidence rates

**Figure 5:**
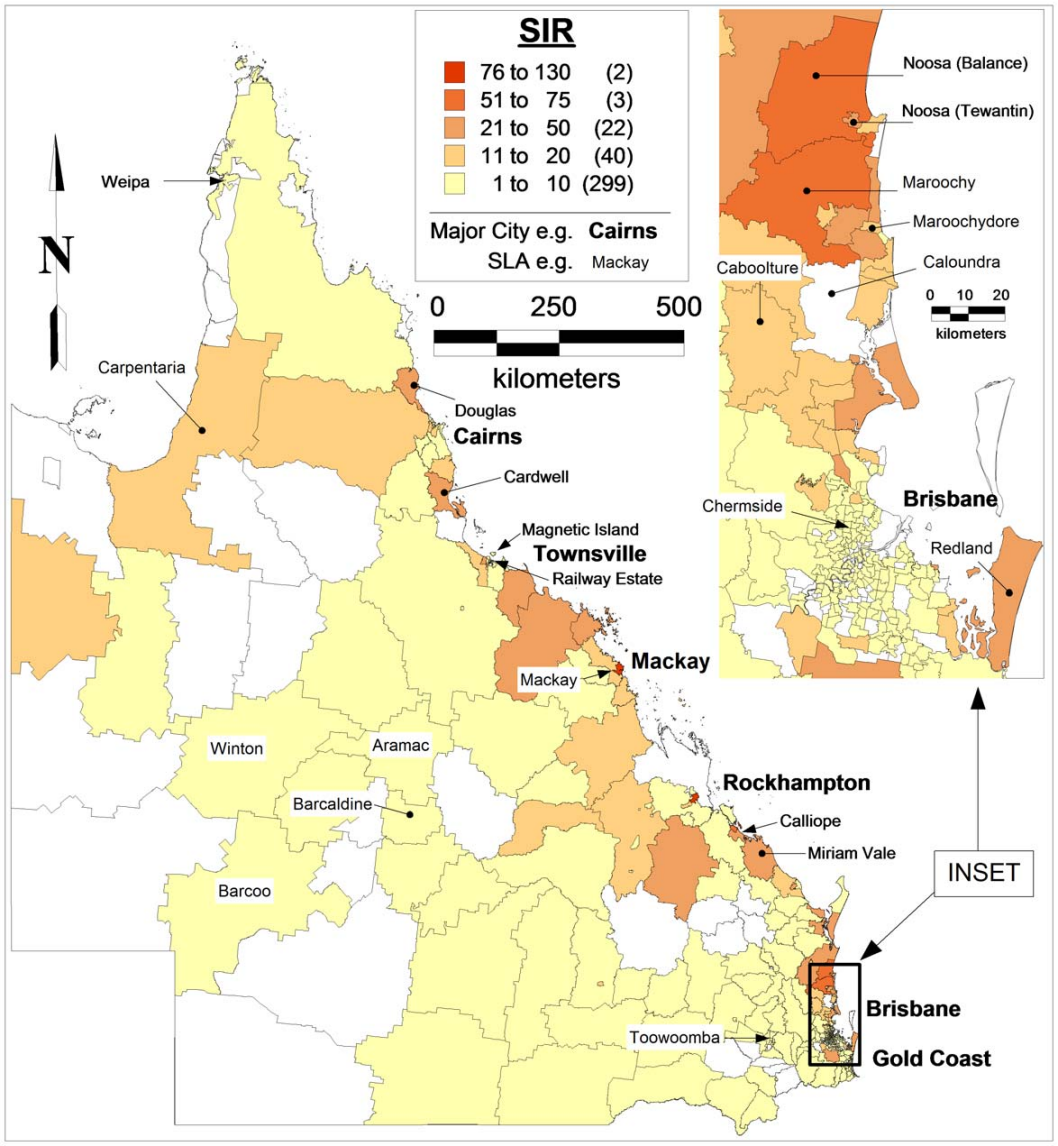
Map showing the standardised incidence rates (1/100,000 people) of BFV disease by SLA in Queensland, 1993–2008.

Standardised incidence rates (SIRs) of BFV disease for each of the SLA in Queensland were calculated and mapped (Fig 5). Geographically, the highest SIRs were observed in the coastal regions; with the peak SIR of 126.9 /100,000 for Mackay while the average SIR in Queensland was 6.09 /100,000. The SIRs were>60/100,000 for 3 SLAs (0.6%);>25 to 50 /100,000 for 13 SLAs (3.5%);>10 to 24.5 /100,000 for 57 SLAs (11%) and >0.2 to 9.9/100,000 for 358 SLAs (74.8%).

Overall, there were statistically significant differences between observed and expected values of BFV disease among SLAs (χ^2^ = 370.0 df = 1,p<0.01). Likewise, statistically significant differences were obtained between observed and expected values of BFV disease among males (χ^2^ = 404.1, df = 1,p<0.01) and females (χ^2^ = 397.0, df = 1434,p<0.01).

Furthermore, spatially significant differences were obtained between the observed and expected values of BFV cases by calculating the confidence interval for each SLA (Table 4).

**Table 4:** SLAs with significant difference between observed and expected values of BFV cases.

SLA name	Mean	SD	CI
Chermside	3.00	0.28	0.45**–**5.54
Toowoomba	5.92	3.36	1.74**–**10.09
Cairns	9.88	5.49	5.29**–**14.48
Caboolture	16.65	6.84	5.76**–**27.53
Caloundra	21.46	16.22	1.31**–**41.60
Maroochydore	29.78	17.72	13.39**–**46.17

#### Semi-variogram analysis and kriging

**Figure 6:**
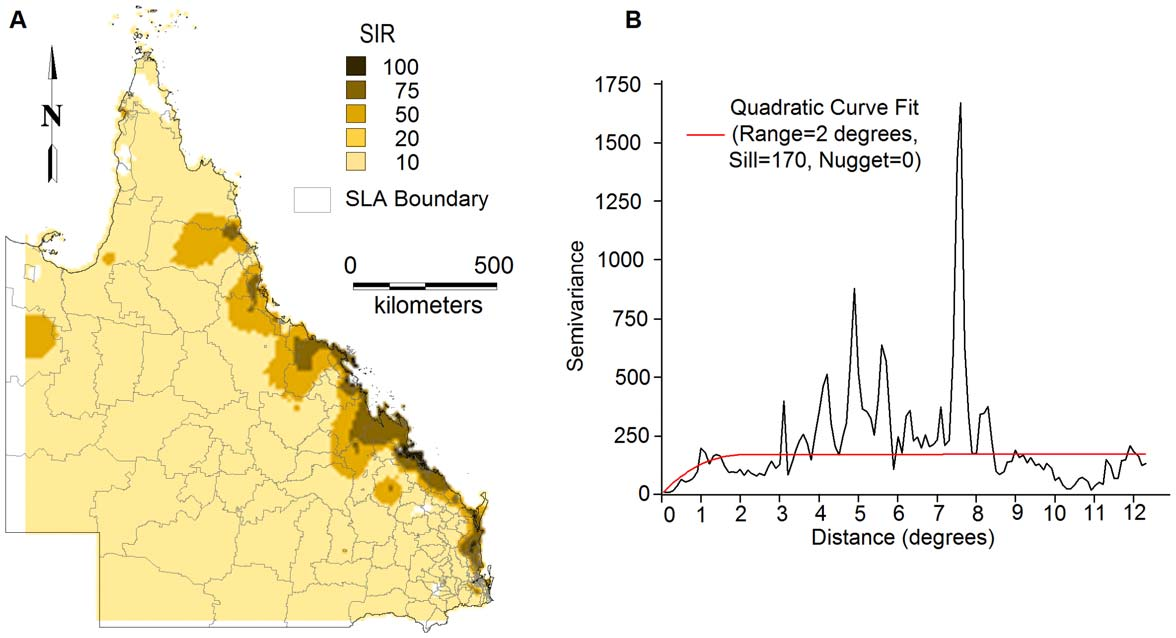
Panel A showing a smoothed map of standardised incidence rates of BFV disease using kriging and panel B showing a semi-variogram model.

Spatial dependence of SIRs was evaluated using semi-variograms. A quadratic model was fitted to the semi-variogram using a sill and nugget of 170 and 0, respectively and a range of 2 degrees (Fig 6). This best-fit semi-variogram model was then used in the kriging procedure to map the SIRs. The map of the kriged SIRs was shown in Figure 6. This shows that the pattern of SIRs of BFV disease is not homogeneous across the state.

Semi-variogram analysis was also undertaken to explore the spatial structure and spatial autocorrelation of age. The semi-variogram showed that there was negligible autocorrelation in age (figure not shown).

## Discussion

This study reveals the spatial and temporal characteristics of BFV disease in Queensland using GIS tools and geostatistical analysis. These methods have been applied to infectious and vector-borne diseases to study the distribution patterns of the disease, to identify the high-risk areas or hot spots, and to determine the risk factors for the transmission of the disease [11], [30], [33], [57], [58]. However, this is the first attempt to implement GIS mapping techniques to examine the distribution of BFV patterns in Queensland and to provide basic information for further investigation of the social and environmental factors responsible for changing disease patterns.

Our findings indicate that the BFV disease transmission occurs in all age groups but mostly affects men aged 40**–**49 years (see Fig 2). The reasons for the higher incidence rates among males are unknown, but may include different exposure rates or other behavioural risk factors such as greater mobility and work and leisure related activities [40], however, this kind of information is unavailable in this study. Clearly, the relationship between the incidence rate of BFV disease and age, and gender needs to be better understood. Further investigation is warranted to determine the underlying differences in exposure or behavioural risk factors to prevent incidence spikes in certain age groups and in men.

The results of this study indicate significant variation in the spatial distribution of BFV disease in Queensland. In this analysis, disease mapping clearly shows that there has been the spatial expansion of BFV transmission in Queensland over recent years. Our results also showed that there has been an increasing trend in incidence rates of BFV disease during the study period. This is in contrast to the patterns observed in other mosquito-borne diseases such as RRV [59].

This study only focused on the spatio-temporal patterns of BFV disease transmission but did not explore its reasons, however, certain speculations could be made. In our study, BFV disease has been detected in all SLAs across the state. Our findings revealed that the coastal regions had the highest incidence rates and SIRs and the inland areas had the lowest (see Figs 4&6). This may be due to the several conditions that favour mosquito density, survival and longevity [3], [4], [60]. A combination of flooding or high tides and heavy rainfall has often resulted in the BFV outbreaks across Australia [61], [62], [63], [64]. In addition, mosquitoes in coastal areas are believed to possess more infections than those in other regions [1], [3], [4], [65]. Therefore, it is evident that further studies on the role of climate on BFV disease mosquitoes would assist in identifying the reasons behind this phenomenal variation. To do this, regional mosquito data would be beneficial; however, such data are scarce in Queensland.

Regarding the temporal distribution of BFV disease, significant differences were noticeable across the state. Our results showed that the annual incidence rates were fluctuated considerably, with the peak incidence rate in 2008 (see Fig 1). These variations may due partly to local changes in climate and human behaviour [4], [8], [27], [66] and partly to under-funded vector control programs [67].

Our results strongly support previous studies that have reported a strong seasonal pattern of BFV disease [4], [27], [31], [68], [69]. In addition, our recent study on time series analysis clearly showed the seasonal patterns of BFV disease [29]. Generally, BFV disease transmission occurs during autumn and summer periods, with peaks recorded during the month of March, immediately after the main rainy season. This is because the mosquito population usually peaks in summer, resulting in a lagged impact on the seasonal variation of BFV disease [4]. Additionally, the geographic distribution of mosquito species and their seasonal activity is mostly determined by climate [70]. Clearly, climate, virus, vector survival and human related factors (eg. behaviour and immunity), all contribute and interact in determining BFV disease transmission. The question about how BFV disease is driven by climatic, socio-demographic and ecological factors will be addressed in further research.

Spatial autocorrelation and semi-variogram analysis are valuable tools to study the spatial patterns over time. The semi-variogram estimators used in this paper directly account for population size, attenuating the influence of less reliable rates recorded in sparsely populated areas. In this study, we found strong evidence of spatial autocorrelation of BFV disease across the state using the global Moran's *I* statistic. Maps created from kriging and interpolation revealed that BFV disease was spatially and temporally distributed. Further studies of local environmental and socio-demographic factors that operate at smaller spatial scales are crucial for improving the understanding of the spatial and temporal patterns of BFV disease. Moreover, further investigation is warranted to understand the effect of climatic and topographic factors on BFV disease transmission in the study area.

There could be issues in monitoring and reporting BFV disease notification data. The clinically proven cases on BFV disease were provided by Queensland Department of Health. BFV disease is one of the notified infectious diseases in Australia, and is required to be reported to the health authority by law. This disease has been under formal surveillance by Australian government since 1993. Issues regarding data reliability were discussed by Russell [71]. However, there are likely to be subclinical cases that are not reported or diagnosed. Underreporting is also likely to occur when people infected with BFV disease but did not seek medical attention. Nevertheless, these issues cannot entirely account for the geographic distribution of BFV disease across Queensland.

Our findings are consistent with the previous studies [54], [72], [73] based on this population. In all these studies, BFV cases were distributed with large variation in each year in Queensland. Our findings are in contrast to the report from Communicable Disease Branch, Queensland Department of Health [59]. The two studies differ in a number of ways. Firstly, our data analysis was based on SLAs and the report was based on Queensland area health services. Secondly, our study population size was calculated based on 2001 and 2006 census years and for the remaining years, it was calculated based on population growth whereas the report was based on estimated resident population [74]. Thirdly, our study period was for the years 1993 to 2008 whereas the report was for 1997–2006 [75]. More importantly, our incidence rates were standardised by age/gender using Queensland total population as the reference, while crude incidence rates were used in the report [75].

This study has three major strengths. Firstly, this is the first study to examine the geographic variation of BFV disease across geo-political borders in Queensland using GIS techniques. This study lays a foundation for further investigation of the spatial and temporal patterns and the risk factors of this disease. Secondly, the results of this study demonstrate that GIS mapping techniques may be used as a tool to quickly display information and generate maps to highlight BFV disease risk-prone areas for developing more effective control and prevention strategies. The maps could be used to suggest high-risk areas where further investigation should be focused, to identify whether increased disease surveillance measures or possible control activities are warranted. Finally, the BFV disease data used in this study are quite comprehensive, covering the whole Queensland for 16 years.

The study has also three key limitations. First, in our analysis, the quality of disease surveillance system may vary with place and time as the awareness of BFV disease among medical professionals and public may have increased over recent years. However, heterogeneity of increased BFV activities suggests that the BFV transmission pattern is unlikely to be entirely accounted for by a detection/surveillance artifact. Second, the locations where BFV cases were notified may differ from those where they caught the disease, particularly during holiday periods, and misclassification bias is inevitable to some extent. Finally, in this study, we aimed to examine the distribution patterns of BFV disease spatially and temporally at the smallest geographical unit of the Australian census, i.e., the SLA level. This study is an ecological design and individual level data are unavailable for this study. Therefore, point-based analysis of the data is beyond the scope of this study.

In conclusion, this study has revealed that the spatio-temporal patterns of BFV disease vary significantly in Queensland as the study has highlighted that there are different transmission patterns in SLAs between coastal and inland regions. The study has also concluded that the geographic distribution of BFV disease appears to have expanded over recent decades. This is based on the results (see Tables 1&2) and on the observation that BFV disease has spread from north to south and west during 1993–2008. The disease maps may be useful for enhanced BFV control activities. There is a lack of knowledge on the transmission dynamics of BFV disease in Australia and this study may help to understand the distribution of BFV disease in Queensland. Future research should focus on the spectrum of risk factors for BFV disease transmission and the development of early warning systems which are necessary to improve the effectiveness and efficiency of BFV prevention and control programs.

## Supporting Information

Appendix S1 An example using actual data from the study sample using direct standardisation method to calculate standardised incidence rates (SIRs) and 95% confidence intervals (CI) of BFV disease only including six SLAs in Queensland is shown in below tables: Table 1(a): Population data by age, gender and total for each SLA. Table 1(b): Number of BFV cases by age, gender and total for each SLA. Table 1(c): Calculation of BFV incidence rate (10^5^) by age, gender and total for each SLA. Table 1(d): Calculation of expected BFV number (BFV incidence/10^5^ divided by total population by age, gender) for each SLA. Table 1(e): Calculation of SIR and CI of BFV incidence rate (/10^5^) for each SLAPopulation data by age, gender and total for each SLA.
(DOC)
