# Supplementary material for: Spatio-Temporal Patterns of Barmah Forest Virus Disease in Queensland, Australia
Source: PLoS One. 2011 Oct 13;6(10):e25688. doi: 10.1371/journal.pone.0025688 (PMC3192738; doi:10.1371/journal.pone.0025688)
Supplement: Appendix S1 — An example using actual data from the study sample using direct standardisation method to calculate standardised incidence rates (SIRs) and 95% confidence intervals (CI) of BFV disease only including six SLAs in Queensland is shown in below tables: Table 1(a): Population data by age, gender and total for each SLA. Table 1(b): Number of BFV cases by age, gender and total for each SLA. Table 1(c): Calculation of BFV incidence rate (105) by age, gender and total for each SLA. Table 1(d): Calculation of expected BFV number (BFV incidence/105 divided by total population by age, gender) for each SLA. Table 1(e): Calculation of SIR and CI of BFV incidence rate (/105) for each SLAPopulation data by age, gender and total for each SLA. (DOC) [file pone.0025688.s001.doc]

**Appendix S1**

An example using actual data from the study sample using direct standardisation method to calculate standardised incidence rates (SIRs) and 95% confidence intervals (CI) of BFV disease only including six SLAs in Queensland is shown in below tables:

Table 1(a): Population data by age, gender and total for each SLA

| SLA name | Male | | | | Female | | | | Total |
| --- | --- | --- | --- | --- | --- | --- | --- | --- | --- |
|  | 40-44 | 45-49 | 50-54 | 55-59 | 40-44 | 45-49 | 50-54 | 55-59 |  |
| A | 281 | 200 | 187 | 184 | 229 | 199 | 218 | 190 | 1688 |
| B | 168 | 188 | 149 | 135 | 164 | 186 | 127 | 129 | 1246 |
| C | 699 | 705 | 535 | 490 | 794 | 714 | 565 | 484 | 4986 |
| D | 79 | 72 | 62 | 65 | 74 | 73 | 68 | 53 | 546 |
| E | 204 | 154 | 159 | 145 | 202 | 166 | 144 | 123 | 1297 |
| F | 660 | 694 | 628 | 550 | 707 | 748 | 649 | 542 | 5178 |
| **Total population** | **2091** | **2013** | **1720** | **1569** | **2170** | **2086** | **1771** | **1521** | **14941** |

Table 1(b): Number of BFV cases by age, gender and total for each SLA

| SLA name |  | Male |  |  |  | Female |  |  | Total |
| --- | --- | --- | --- | --- | --- | --- | --- | --- | --- |
|  | 40-44 | 45-49 | 50-54 | 55-59 | 40-44 | 45-49 | 50-54 | 55-59 |  |
| A | 1 | 1  2  0  0  1  1  **5** | 0 | 0 | 1  0  3  0  0  3  **7** | 0 | 0 | 0 | 3 |
| B | 0 |  | 0 | 1 |  | 0 | 1 | 1 | 5 |
| C | 1 |  | 0 | 0 |  | 2 | 1 | 0 | 7 |
| D | 0 |  | 0 | 0 |  | 0 | 0 | 0 | 0 |
| E | 0 |  | 2 | 0 |  | 1 | 0 | 0 | 4 |
| F | 3 |  | 1 | 1 |  | 1 | 2 | 0 | 12 |
| **Total BFV cases** | **5** |  | **3** | **2** |  | **4** | **4** | **1** | **31** |

Table 1(c): Calculation of BFV incidence rate (10^5^) by age, gender and total for each SLA

| SLA name | Male | | | | Female | | | | Total |
| --- | --- | --- | --- | --- | --- | --- | --- | --- | --- |
|  | 40-44 | 45-49 | 50-54 | 55-59 | 40-44 | 45-49 | 50-54 | 55-59 |  |
| A | 355.9 | 500.0 | 0 | 0 | 436.7 | 0 | 0 | 0 | 1292.6 |
| B | 0 | 1063.8 | 0 | 740.7 | 0 | 0 | 787.4 | 775.2 | 3367.1 |
| C | 143.1 | 0 | 0 | 0 | 377.8 | 280.1 | 177.0 | 0 | 978 |
| D | 0 | 0 | 0 | 0 | 0 | 0 | 0 | 0 | 0 |
| E | 0 | 649.4 | 1257.9 | 0 | 0 | 602.4 | 0 | 0 | 2509.7 |
| F | 454.5 | 144.1 | 159.2 | 181.8 | 424.3 | 133.7 | 308.2 | 0 | 1805.8 |
|  | **953.5** | **2357.3** | **1417.1** | **922.5** | **1238.8** | **1016.2** | **1272.6** | **775.2** | **9953.2** |

Table 1(d): Calculation of expected BFV number (BFV incidence/10^5^ divided by total population by age, gender) for each SLA

| SLA No | SLA Name | Male | | | | Female | | | | Total |
| --- | --- | --- | --- | --- | --- | --- | --- | --- | --- | --- |
|  |  | 40-44 | 45-49 | 50-54 | 55-59 | 40-44 | 45-49 | 50-54 | 55-59 |  |
| 31001 | A | 15 | 0 | 0 | 0 | 13 | 18 | 0 | 0 | 46 |
| 37001 | B | 0 | 0 | 20 | 20 | 0 | 28 | 0 | 19 | 87 |
| 35951 | C | 31 | 23 | 15 | 0 | 12 | 0 | 0 | 0 | 81 |
| 31004 | D | 0 | 0 | 0 | 0 | 0 | 0 | 0 | 0 | 0 |
| 31007 | E | 0 | 16 | 0 | 0 | 0 | 17 | 32 | 0 | 65 |
| 36251 | F | 37 | 12 | 27 | 0 | 40 | 13 | 14 | 16 | 158 |
| **Total BFV cases** | | **84** | **51** | **62** | **20** | **64** | **75** | **46** | **35** | **437** |

Table 1(e): Calculation of SIR and CI of BFV incidence rate (/10^5^) for each SLA

| SLA Name | SIR | 95% CI |
| --- | --- | --- |
| A | 1.8 | 15-77 |
| B | 7.6 | 35-139 |
| C | 7.4 | 66-96 |
| D | 0.3 | 0-0 |
| E | 3.0 | 19-111 |
| F | 5.2 | 138-178 |
